# Across-shelf distribution of blue mussel larvae in the northern Gulf of Maine: consequences for population connectivity and a species range boundary

**DOI:** 10.1098/rsos.150513

**Published:** 2015-12-02

**Authors:** Philip O. Yund, Charles E. Tilburg, Michael A. McCartney

**Affiliations:** 1 The Downeast Institute, Beals, ME 04611, USA; 2 Department of Marine Sciences, University of New England, Biddeford, ME 04005, USA; 3 Department of Biology and Marine Biology, University of North Carolina, Wilmington, NC 28409, USA

**Keywords:** across-shelf mixing, range boundary, population connectivity, blue mussel, larval transport

## Abstract

Studies of population connectivity have largely focused on along-shelf, as opposed to across-shelf, processes. We hypothesized that a discontinuity in across-shelf mixing caused by the divergence of the Eastern Maine Coastal Current (EMCC) from shore acts as an ecological barrier to the supply of mussel larvae to the coast. Existing data on the relative abundance of two congeneric blue mussels, *Mytilus edulis* and *M. trossulus*, were analysed to quantify the association of *M. trossulus* with the colder temperature signal of the EMCC and generate larval distribution predictions. We then sampled the across-shelf distribution of larvae along two transects during 2011. Larvae were identified using restriction digests of PCR amplicons from the mitochondrial 16S rDNA. *Mytilus edulis* larvae were consistently abundant on either the inshore and offshore transect ends, but not homogeneously distributed across the shelf, while *M. trossulus* larvae were less common throughout the study area. The divergence of the EMCC from shore appears to create a break in the connectivity of *M. edulis* populations by isolating those inshore of the EMCC from upstream larval sources. Across-shelf transport processes can thus produce connectivity patterns that would not be predicted solely on the basis of along-shelf processes.

## 1. Introduction

Most past work on the across-shelf transport of the planktonic larvae of coastal benthic invertebrates has taken place in the context of understanding spatial and temporal variation in onshore settlement. Consequently, the mechanisms responsible for returning larvae to shore, including upwelling [1], relaxation of upwelling [2], across-shelf winds [3–5], downwelling [6,7], tides [8] and internal waves [2,9,10], have been well documented. Statistical relationships between larval settlement and across-shelf transport processes provide particularly strong support for many of these mechanisms [11–14].

In contrast to these studies—motivated primarily by understanding settlement variation—most work on larval dispersal in a population connectivity context has focused on the mechanisms involved in along-shelf larval transport [6,15–20]. Currents along the shelf are typically stronger and, therefore, easier to measure than across-shelf flows [21], so this emphasis partly reflects strengths and weaknesses in our current understanding of coastal circulation [22]. As a consequence of this along-shelf emphasis, across-shelf transport processes have not been well integrated into connectivity studies [22–24]. In coupled biophysical transport models, competent larvae are often assumed to settle successfully if they simply arrive within a specified distance of suitable shore habitat (e.g. 9–18 km [16,20]). Alternatively, a variety of across-shelf mixing scenarios may be modelled in the absence of information on actual across-shelf transport [17].

Interfaces between water masses represent zones of reduced mixing that may impede larval dispersal across a frontal boundary [25–28]. Past studies have examined the effects of fronts associated with upwelling events [14,25,26], major shelf current systems [27], slope currents [29], tidal intrusions [30] and very near-shore (0.1–1 km scale) coastal boundary layers [24,31]. In coastal systems, another potentially common scenario involves an along-shelf coastal current that diverges from shore due to bathymetric steering [32–34]. Limited mixing between an along-shelf current and inshore waters throughout a region of divergence can potentially reduce the supply of larvae to the coast inshore of that current and cause a break in population connectivity [28].

We hypothesize that such a scenario occurs in the northern Gulf of Maine, where the southwestward-flowing Eastern Maine Coastal Current (EMCC) loosely follows the 75 m isobath offshore [32]. The EMCC starts to diverge from shore at Machias Bay in far eastern Maine and this divergence creates a near-shore zone of flow and temperature fields [35] that are distinctly different from those within the EMCC. As part of an earlier paper [28], we presented an initial dataset showing that the distribution of bivalve larvae along three across-shelf transects in the EMCC region on four different sampling dates in 2010 was consistent with the hypothesis of limited across-shelf mixing. Here, we expand on this earlier effort in three important ways. First, we re-analysed previously published data from 2001 and 2005 [36,37] to show that adult populations of the northern blue mussel, *Mytilus trossulus*, are strongly associated with the colder waters of the EMCC. Second, we sampled two of the transects studied in 2010 throughout a full season of mussel larval dispersal in 2011, allowing us to assess temporal as well as spatial patterns. And third, we identified the collected *Mytilus* blue mussel larvae (*M. edulis* and *M. trossulus*) to the species level. Because these two species have different adult distributions within the region, larval sources should also differ, with larvae of *M. trossulus* expected to originate in upstream populations and to be mostly delivered to the study region via the EMCC. By contrast, because *M. edulis* populations are present both inshore of the EMCC and in upstream regions, those larvae (in the absence of across-shelf mixing) should be abundant at both ends of the transects, but not homogeneously distributed along the transects. Hence, sampling larvae of these two species provides a stronger test of the limited across-shelf mixing hypothesis than our earlier limited analysis of bivalve larvae from multiple unidentified genera [28].

## 2. Material and methods

### 2.1 Coastal circulation in the study region

The coastal circulation along the western boundary of the Gulf of Maine is dominated by two coastal currents with intermittent interaction [32,38,39]: the Western Maine Coastal Current (WMCC) and the EMCC. Our study site encompasses a region in the northern Gulf of Maine where the location of the southwestward-flowing EMCC follows the 75 m isobath offshore starting around Machias Bay [32] but shifts position with upwelling and downwelling winds [39]. Further to the southwest (Penobscot Bay), the EMCC either turns offshore and contributes to the Jordan Basin cyclonic gyre [32] or turns back shoreward to merge into the WMCC, which flows in close proximity to shore [40,41]. The region inshore of the EMCC is characterized by warmer, slightly fresher water that results in strong vertical stratification and horizontal temperature gradients [28,35].

### 2.2 Distribution of adult mussels

**Figure 1.**
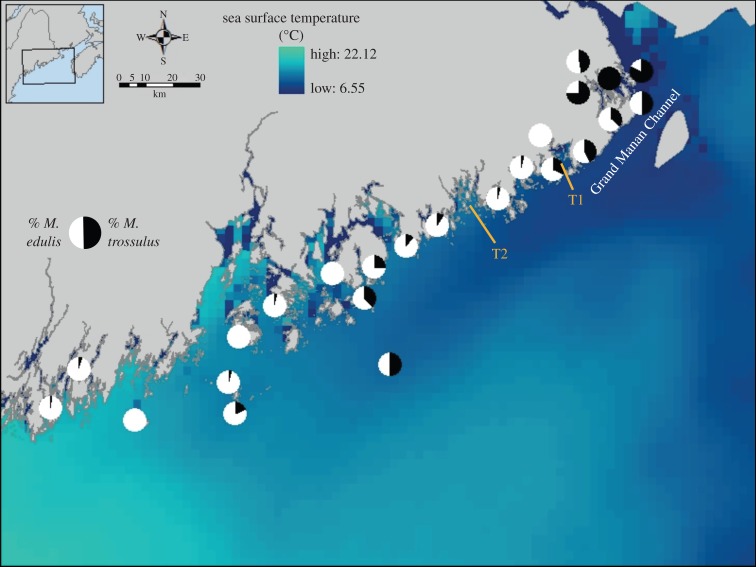
Distribution of *M. edulis* and *M. trossulus* in Eastern Maine. Pie charts depict the relative abundance of the two species (data re-plotted from [37]). The ocean background depicts the 25-year SST climatology for the month of June (data from the University of Maine’s Satellite Oceanography Data Laboratory—http://wavy.umeoce.maine.edu). The EMCC is apparent as the southwestward-flowing tongue of colder water that diverges from the coast. We sampled the across-shelf distribution of larvae of the two mussel species along the two transects (T1 and T2) represented by orange lines.

The sibling blue mussel species *M. edulis* and *M. trossulus* are largely indistinguishable on morphological criteria but can be reliably distinguished with genetic markers [42–44]. Previous work by Rawson and co-workers [36,37] used a pair of diagnostic loci to map the relative abundance of adults of the two species in eastern Maine during 2001 and 2005 and noted that the range boundary of *M. trossulus* corresponded to the divergence of the EMCC from the coast. The EMCC is easily recognized in satellite sea surface temperature (SST) data as a tongue of cold water that extends southwestward from the Grand Manan Channel and offshore. To quantitatively assess the association between adult *M. trossulus* and the EMCC, we first plotted previously published relative abundance data (% composition of the mussel population) from Hayhurst & Rawson [37] on a background of the 25-year satellite SST climatology for the month of June obtained from the University of Maine’s Satellite Oceanography Data Laboratory (http://wavy.umeoce.maine.edu/). We selected June because this is the month in which larval dispersal commences. Consequently, temperature patterns can be used both to evaluate the temperatures experienced by larvae in the water column and to visualize the location of sites with respect to the EMCC during the critical period of dispersal. Temperatures in other months may impact the adult populations, but our goal was to test for a quantitative relationship with the EMCC temperature signal during the dispersal period. Relative abundance data were based on the species-specific Glu-5^′^ locus, but a second diagnostic locus (ITS) yielded a virtually identical distribution pattern [37]. Second, we quantified the association with the EMCC temperature signal by regressing relative *M. trossulus* abundance on the local temperature climatology. Sites with relative abundance data were matched with geo-referenced SST climatology values (represented as specific pixels in figure 1). If no valid temperature value was available for the actual coastal location where mussels were collected (due to shoreline effects in the SST algorithm), the adjacent offshore SST value (within 1 km) was substituted as a proxy.

### 2.3 Field sampling

The across-shelf distributions of *M. edulis* and *M. trossulus* larvae were sampled along two transects in 2011. Transects originated in Machias Bay (T1 in figure 1) and Pleasant Bay (T2 in figure 1) and were oriented perpendicular to the shelf and the flow of the EMCC. Transect 1 (Machias Bay) comprised four stations (table 1) distributed at approximately equal distances along its 10 km length, while transect 2 (Pleasant Bay) had six stations (table 1) distributed at similar intervals (3–6 km) along its greater 24 km length. We conducted eight sampling cruises, with one cruise occurring every other week throughout the mussel larval dispersal season (approximately mid-May through early September).

**Table 1.** Coordinates of sampling stations. Station numbers increase from inshore to offshore.

transect	station	latitude °N	longitude °W
T1	1	44.65741	67.34995
T1	2	44.61862	67.32780
T1	3	44.58505	67.32554
T1	4	44.56540	67.31016
T2	1	44.50559	67.77594
T2	2	44.48253	67.76675
T2	3	44.45480	67.75105
T2	4	44.42652	67.72467
T2	5	44.37468	67.67178
T2	6	44.33746	67.63731

On each cruise, we used an impeller-driven water pump to collect larvae contained in 100 l of water at 5 m depth at each station on the two transects. Bivalve larvae are quite rare in surface waters in this region, but are abundant at 5 m depth [28]. *Mytilus* spp. larvae tend to be distributed in the upper 8 m of the water column [45] and settle mainly near the surface [46,47], but potentially migrate vertically in response to tides [48], phytoplankton abundance and haloclines [49]. The 5 m depth was intended to represent approximately the mid-point of the likely vertical distribution across a range of conditions. Three replicate samples at each station were filtered through a 50 μm plankton net and immediately preserved in modified salt ethanol (MSE) [50]. Samples were processed under a dissecting microscope and all bivalve veligers were manually sorted from the remainder of the sample, enumerated and stored in MSE for subsequent genetic identification. Earlier stage trochophore larvae were rare in our samples (they are likely to be more fragile than later stages) and were not quantified. Concurrent with larval sample collection, the physical hydrography of the water column at each site was characterized by deploying a conductivity–temperature–depth instrument (CTD; Seabird SBE-25).

### 2.4 Genetic identification of larvae

The proportion of *M. edulis* and *M. trossulus* larvae in samples was calculated by extracting bulk DNA from the mixed field samples, amplifying the 16S gene, cloning the amplicons into bacteria and then performing restriction digests of 16S products re-amplified from multiple clones. First, the sorted bivalve samples were processed a second time to carefully exclude any contaminating zooplankters that could potentially amplify with our universal 16S primers and interfere with our ability to quantify the relative abundance of bivalve larvae of different species. One hundred to 200 bivalve larvae were removed from samples with more than 100 bivalve larvae in total; all larvae were picked from samples with less than 100 larvae, and stations with very few bivalve larvae present (mean of less than 10 larvae per replicate) were not processed. Larvae were washed in 70% ethanol and then combined together in a 0.2 ml PCR tube. Ethanol was removed by pipetting and evaporating at 65°C for 20 min and samples were stored at −80°C until they were extracted. DNA was extracted by adding 15 μl of larval extraction buffer (100 μl of MyTaq Red Buffer (Bioline, Taunton MA) +10 μl Tween 20+50 μl proteinase K (25 mg ml^−1^) in 1.0 ml total) to the sample and sonifying larvae for 30 s at 60 amps with a microprobe. Finally, the extract was heated at 65°C for 90 s, followed by 95°C for 15 s.

PCR of 1 μl of extract was performed in 25 μl total volume (5 μl MyTaq Red Buffer+1.25 μl 10 μM primers 16sar-L/16sbr-H [51] +0.25 μl MyTaq HS (Bioline)). Thermal cycler conditions were 94°C for 3 min followed by 35 cycles of 94°C for 1 min, 47°C for 1 min and 72°C for 1 min; followed by 72°C for 45 min. Amplicons were purified over Strataprep columns (Agilent, Santa Clara, CA, USA) and the eluate was concentrated by vacuum evaporation and cloned into Promega T-easy vector (Madison, WI, USA). One hundred clones were picked and each clone was lysed by heating for 5 min in 20 μl ddH_2_O. Clone lysates were re-amplified using 16S primers (digests of products amplified using vector primers were more difficult to interpret) and PCR products were triple-digested using *Hae* III, *Spe* I and *Eco* RV endonucleases, using a modification of methods provided in Rawson & Hilbish [42]. A minimum of 48 clones were amplified and the digested products were sized on 14×23 cm 1.5% Metaphor agarose gels (Lonza, Allendale, NJ, USA). Diagnostic digestion profiles for *M. edulis* and for *M. trossulus* were confirmed by sequencing. All clones were scored as representing one of the *Mytilus* species or an ‘other’ category that matched neither fragment pattern. The proportional sample composition obtained via this method was then multiplied by the original total bivalve count to estimate the number of each *Mytilus* species in each sample, subject to a minor modification described below.

We tested this identification approach by creating artificial mixtures of larvae of the two *Mytilus* species. Larvae were produced from laboratory crosses of *M. edulis*×*M. edulis* and *M. trossulus*×*M. trossulus* following the adult identification and fertilization methods of Slaughter *et al.* [52]. Each of the 10 mixtures consisted of 200–210 larvae with species ratios of 20:1, 19:1, 9.5:1, 9:1, 4:1 (two replicates) and 0:1 (2 replicates of both of the two possible combinations). Control mixtures were extracted and analysed using methods identical to those described above for field samples, with the exception that 96 clones were analysed from each control mixture.

### 2.5 Larval data analysis

Data from the control mixtures were analysed by regressing the estimated proportion of the *Mytilus* species on the known proportion in each mixture. Because of a slight nonlinearity to the relationship (see Results), we fitted a third-order polynomial to the data. This equation was then used to adjust the raw proportions obtained for the field samples and had the effect of very slightly increasing the proportion of the rarer species (almost always *M. trossulus*). Larval concentrations were plotted on a background of temperature data from the 5 m collection depth, with time (cruise) constituting the *x*-axis and location along the transect constituting the *y*-axis. Larval concentrations of both species along each transect on each cruise were analysed with one-way ANOVAs to assess possible station effects. A Tukey’s post hoc test was used to assess differences among individual stations.

Samples from stations with a mean of less than 10 total bivalve larvae were not identified to the species level and for both visualization and statistical purposes were inferred to represent a concentration of 0 larvae for both mussel species. The majority of un-processed replicates from these stations contained literally 0 bivalve larvae, but the station was classified in this category if even one replicate from that cruise/station combination contained a small non-zero number of larvae. On average, these unidentified samples contained 3.3±0.6 (s.e.) bivalve larvae constituting some unknown mixture of larvae of *M. edulis*, *M. trossulus* and all other bivalve taxa present. Our genetic technique was not designed to accommodate such very low larval concentrations (other station/date combinations typically contained in the range of hundreds to thousands of larvae) and resolving the species composition of these very small samples was deemed irrelevant to assessing spatial and temporal patterns. These inferred zeros typically occurred early in the season and are noted in the presentation of results so that readers may assess any possible impact on interpretation.

The strong association between adult *M. trossulus* and the temperature signal of the EMCC (see Results) permitted us to generate a set of expectations for the across-shelf distribution of larvae of the two species. We expected *M. trossulus* larvae that reached our transects to originate almost entirely from upshelf adult populations within the EMCC (far eastern Maine and New Brunswick) or on the Scotian shelf. If this assumption was valid and across-shelf mixing was minimal, *M. trossulus* larvae should be more abundant on the offshore portions of our across-shelf transects. Because a few *M. trossulus* adults were present in populations inshore of the EMCC (see Results), we did expect some *M. trossulus* larvae in near-shore waters. However, given limited mixing, *M. trossulus* larvae should constitute a minority, consistent with their frequency in the adult population (*ca* 5–10%; see Results). By contrast, *M. edulis* larvae should originate from populations inshore and within the EMCC, as well as farther upshelf. Consequently, we expected them to be abundant on either or both ends of the across-shelf transects. However, in the absence of across-shelf mixing, *M. edulis* larvae should not be homogeneous across the shelf but should be concentrated towards one or both of the transect ends.

## 3. Results

### 3.1 Distribution of adult mussels

**Figure 2.**
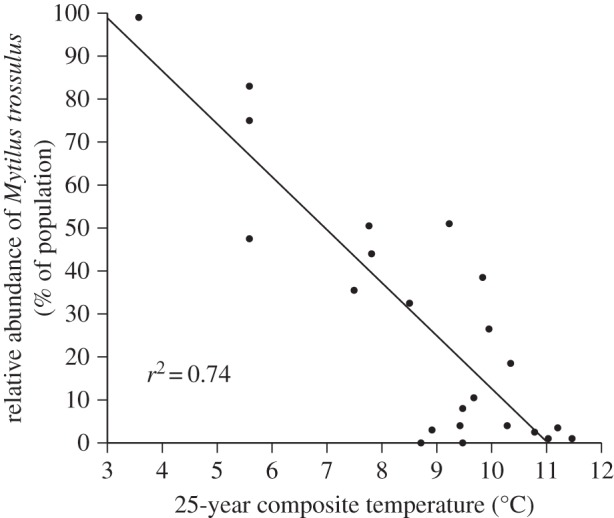
Association of *M. trossulus* with the temperature signal of the EMCC. The relative abundance of *M. trossulus* was regressed on the 25-year SST climatology data from figure 1. Average SST explains 74% of the variance in the relative abundance of the northern mussel species.

Although there was some variation among sites, *M. trossulus* and *M. edulis* were approximately equally abundant in populations in far eastern Maine (figure 1; in the Grand Manan Channel and near the US/Canada boundary) and previous work has shown that this pattern extends north and eastward into Canada [53–55]. As previously noted [37], the coastal range boundary of *M. trossulus* corresponds with the point where the EMCC starts to diverge from shore (figure 1, Machias Bay, the location of T1). Although *M. trossulus* were relatively rare at coastal sites southwest of this bay, their relative abundance was high on offshore islands located within the EMCC (figure 1). A quantification of this association indicates that average June water temperature (the month when most larval dispersal commences), as represented by the 25-year June SST climatology, explains 74% of the variance in *M. trossulus* relative abundance (figure 2).

**Figure 3.**
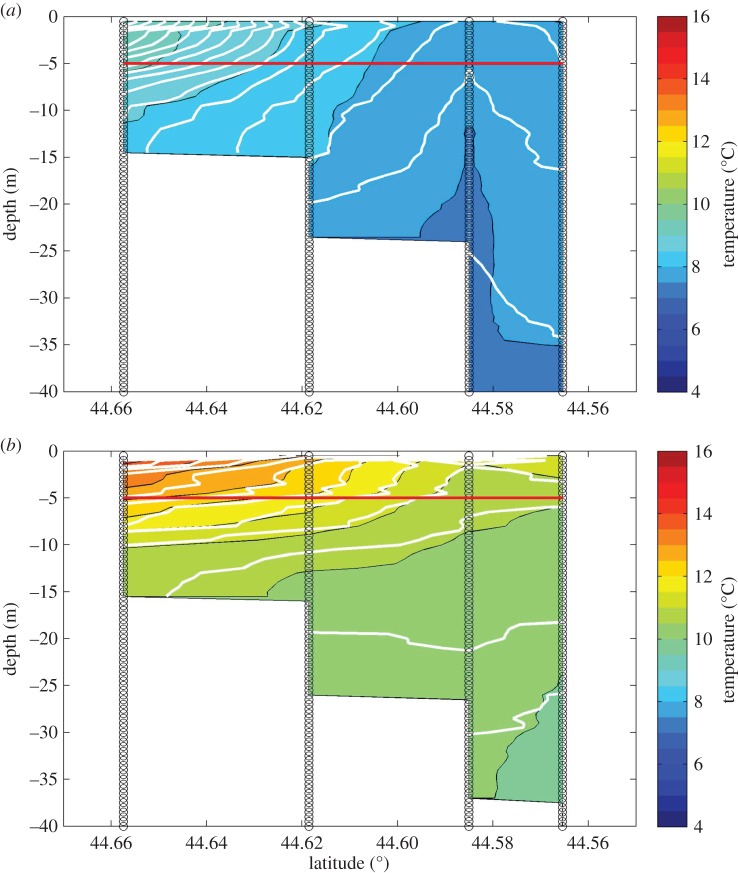
Across-shelf hydrography on transect 1 (*a*) and transect 2 (*b*) during cruise 3, 2011. The red line indicates the depth at which 5 m larval samples were collected. Vertical black lines represent the locations of CTD and larval sampling stations, colour gradations represent water temperatures, and the white contour lines represent isopycnals.

**Figure 4.**
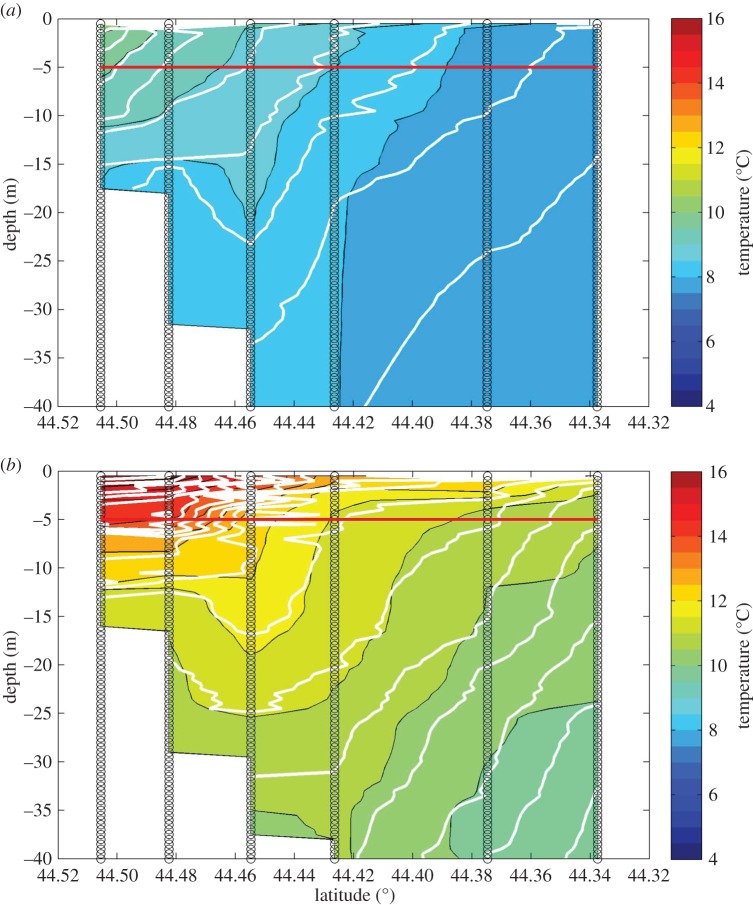
Across-shelf hydrography on transect 1 (*a*) and transect 2 (*b*) during cruise 6, 2011. The red line indicates the depth at which 5 m larval samples were collected. Vertical black lines represent the locations of CTD and larval sampling stations, colour gradations represent water temperatures, and the white contour lines represent isopycnals.

Because the SST patterns in figure 1 represent a 25-year average, they fail to clearly delineate the inshore margin of the EMCC, which fluctuates over time with wind direction [39]. On any given day, the inshore interface is typically defined by a much sharper SST discontinuity [28]. The apparent continuous across-shelf SST gradient in figure 1 is an artefact of using the 25-year composite temperature and should not be interpreted as evidence of across-shelf mixing. The temperature signal of the EMCC is distinct from that of the near-shore waters along transect 1 and at locations southwestward throughout our study area [28]. Reconstructions of the cross-sectional temperature profiles for four cruises in 2011 reveal the strong SST discontinuity (figures 3 and 4). A highly stratified water column on the inshore end of both transects 1 and 2 that transitioned to a more weakly stratified and colder water column on the offshore end is apparent on both transects throughout the study period (figures 3 and 4). The weaker vertical stratification and colder temperatures are typical of the vertically well-mixed EMCC [28,32,39], while the stronger vertical stratification of the near-shore region [34] is consistent with shallower waters affected by freshwater sources. Although the overall temperatures indicate warming between cruises (cf. figures 3*a* and 4*a* with figures 3*b* and 4*b*), the difference between the inshore region and the EMCC is still apparent.

### 3.2 Efficacy of genetic larval identification method

**Figure 5.**
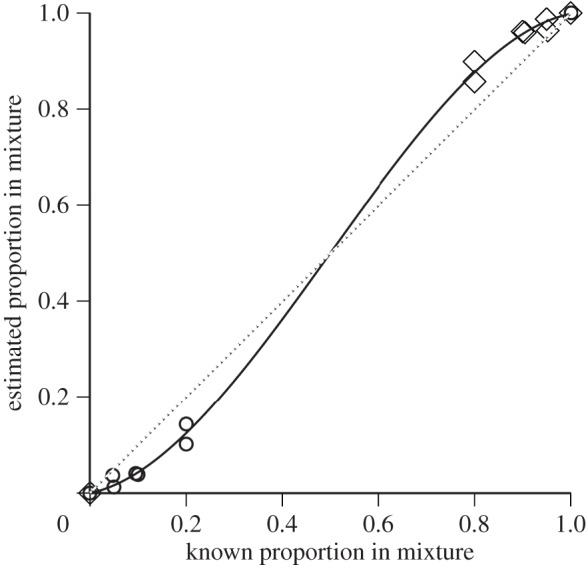
Validation of the cloning-based method used to assess species-specific larval abundance in water column samples. Circles represent *M. edulis* and diamonds represent *M. trossulus.* Artificial mixtures of *M. edulis*and *M. trossulus* larvae of known parentage were quantified as described in the text and the estimated mixture regressed on the known mixture. This technique is very sensitive and can detect larvae that make up only 1–2% of the sample. However, the relationship is slightly nonlinear, with the less abundant species slightly under-represented in the estimated proportions when rare. The third-order polynomial fit to the data was used to adjust raw water column concentration values. Dotted line represents a perfect 1:1 correspondence between known and predicted values. Some symbols for the two species overlap one another.

In artificial mixtures of *M. edulis* and *M. trossulus* larvae, our 16S clone-based quantification method was extremely effective at detecting larvae, even when quite rare (5% of the mixture), and produced relative abundance estimates that were accurately predicted (*r*^2^=0.98) by the known mixture values (figure 5). However, the relationship between the estimated and known proportions was somewhat nonlinear, with our technique slightly underestimating the relative abundance of the rarer species and overestimating the abundance of the more common species (figure 5). A third-order polynomial $P_{A}=1.959P_{O}^{3}-2.981P_{O}^{2}+2.015P_{O}$, where *P*_A_ and *P*_O_ are the actual and observed proportions of larvae, respectively, provided the best fit to the relationship, with a random distribution of residuals. This equation was used to adjust the raw proportion values obtained for field samples.

### 3.3 Across-shelf distribution of larvae and temperature

**Figure 6.**
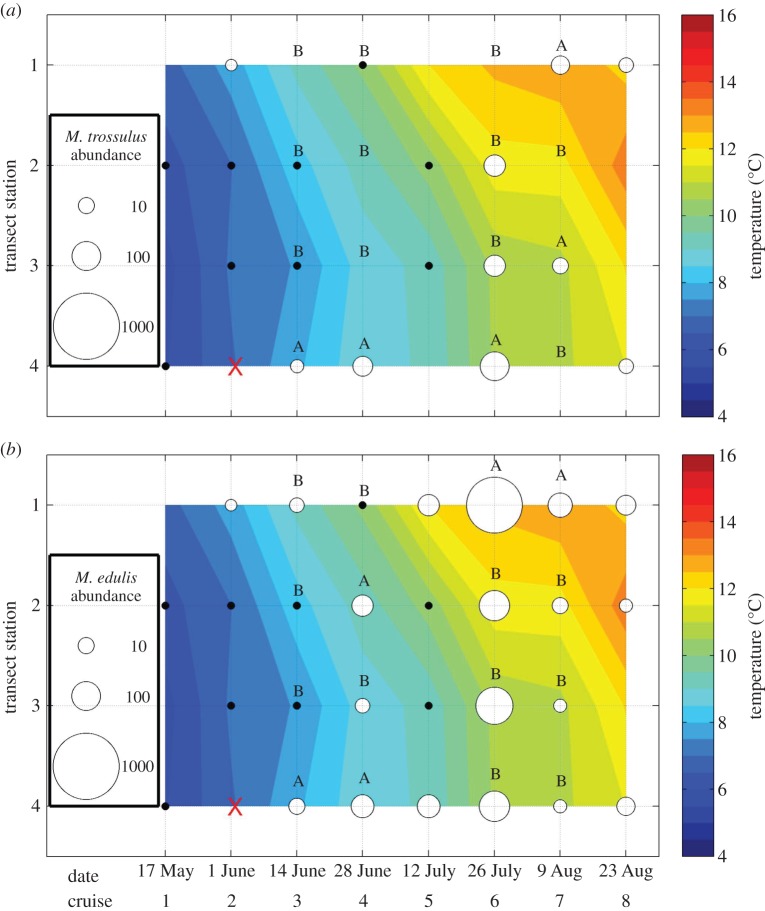
Temporal variation in spatial patterns of larval abundance on transect 1 for (*a*) *M. trossulus*and (*b*) *M. edulis.* Circles are proportional to larval density at 5 m, red ‘X’s represent missing values, and black dots represent inferred zeros from samples with very few larvae. Stations (*y*-axis) are numbered sequentially from onshore to offshore. The background depicts water temperatures at 5 m depth. Letters indicate stations on each transect that did not differ significantly in larval concentration.

**Figure 7.**
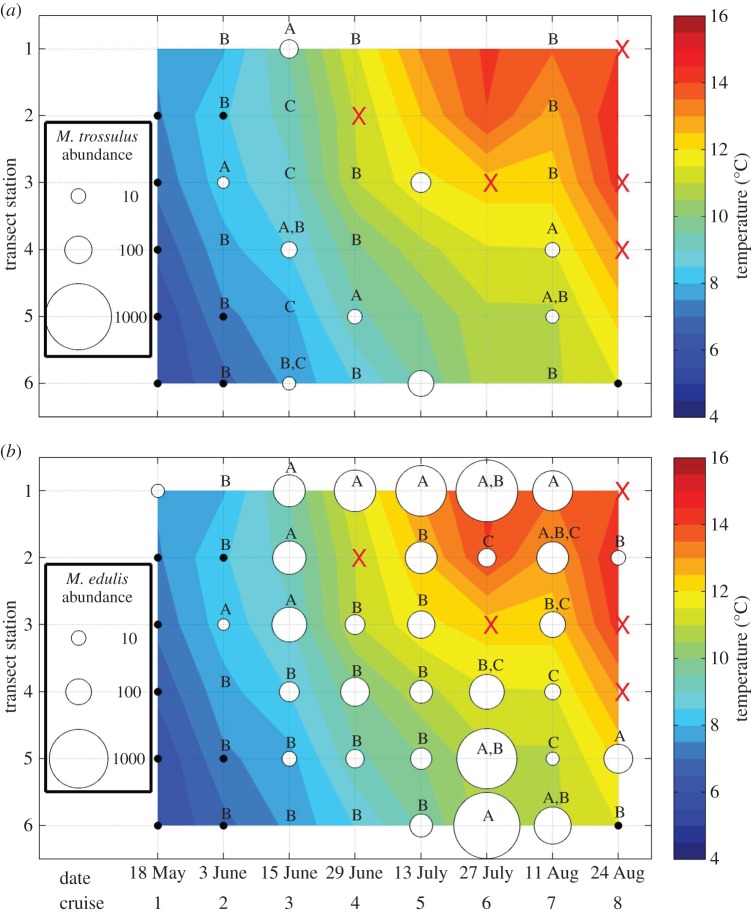
Temporal variation in spatial patterns of larval abundance on transect 2 for (*a*) *M. trossulus* and (*b*) *M. edulis.* Circles are proportional to larval density at 5 m, red ‘X’s represent missing values, and black dots represent inferred zeros from samples with very few larvae. Stations (*y*-axis) are numbered sequentially from onshore to offshore. The background depicts water temperatures at 5 m depth. Letters indicate stations on each transect that did not differ significantly in larval concentration.

Water temperatures at the 5 m larval collection depth were fairly homogeneous along transect 1 on the first cruise (figure 6) but showed a distinct gradient on transect 2 with colder water present offshore (figure 7). All subsequent cruises showed a clear temperature gradient on both transects, with temperatures at the inshore stations generally higher, warming more quickly during the year and reaching higher maxima than the offshore stations (figures 6 and 7). Data from transect 2 showed a slight reversal of the seasonal warming trend on cruise 7 (figures 6 and 7), but overall temperature patterns were consistent with seasonal warming and the development of a stronger across-shelf temperature gradient due to preferential warming of the shallower, near-shore waters.

*Mytilus* larvae were rare on both transects during the first two cruises and then steadily increased through approximately cruise 6 (late July) before declining later in the season (figures 6 and 7). Seasonal presence/absence patterns were very similar for the two species on transect 1, with the first appearance inshore (station 1) on cruise 2 and first offshore (station 4) appearance on cruise 3 (figure 6). By contrast, on transect 2 (figure 7), *M. edulis*first appeared inshore (station 1) on cruise 1, while *M. trossulus* did not appear inshore until cruise 3. The pattern was reversed offshore (station 6), with *M. trossulus* first present on cruise 3, while *M. edulis* larvae were not present until cruise 5 (figure 7). Larvae of both species were rare by the end of the season (cruise 8; figures 6 and 7). We hesitate to infer too much from these seasonal patterns because they are likely to reflect some complex function of different spawning times in multiple source populations and environment-specific larval dispersal and survival rates. Nevertheless, at least one comparison is striking—no *M. trossulus* larvae were present during the period of peak inshore (station 1) *M. edulis* larval abundance on either transect (figures 6 and 7).

The across-shelf distribution of larvae of both species was generally homogeneous only in ecologically trivial cases in which larvae were absent or rare along an entire transect, with such cases occurring early in the season (transect 1, cruises 1 and 2, both species; transect 2, cruise 1, both species; figures 6 and 7), at the very end of the season (transect 1, cruise 8, both species; transect 2, cruise 8, *M. trossulus* only; figures 6 and 7) and during a temporary mid-season drop in larval abundance (cruise 5, transect 1, both species; cruise 6, transect 2, *M. trossulus* only; figures 6 and 7). There was also no station effect on *M. trossulus* larval concentration on transect 2 during one cruise (no. 5) in which larvae were present in high numbers, although there was a trend towards high *M. trossulus* abundance at both the offshore and at one intermediate station, with zero larvae present at the other stations (figure 6*a*). This pattern was not statistically significant because of unusually high variance among the replicate samples at both stations where larvae were present.

The across-shelf distribution of larvae was non-homogeneous for both species on all other transect and cruise combinations. *Mytilus trossulus* larvae were more abundant on the offshore end of transect 1 on cruises 3, 4 and 6 (ANOVAs, respectively; *F*=85.12, *p*<0.0001; *F*=543.51, *p*<0.0001; *F*=18.89, *p*<0.005; all post hoc tests reported in figure 6*a*). During cruise 7, abundance maxima on transect 1 occurred both at the inshore station and at one intermediate station (ANOVA, *F*=10.30, *p*<0.004 and post hoc tests reported in figure 6*a*). Distribution patterns on transect 2 were more complex, with abundance maxima at one or more intermediate stations (cruise 2, ANOVA, *F*=33.22, *p*<0.0001; cruise 4, ANOVA, *F*=49.44, *p*<0.0001; cruise 7, ANOVA, *F*=4.99, *p*<0.01; all post hoc tests reported in figure 7*a*) or distributed in a more multi-modal pattern involving high abundance at both ends of the transect plus an intermediate station (cruise 3, ANOVA, *F*=14.16, *p*<0.0001 and post hoc tests reported in figure 7*a*). In two of the cruises with high *M. trossulus* abundance at one or more intermediate stations (cruises 4 and 7), the relevant stations were located on the EMCC side of the transect (figure 7*a*). *Mytilus trossulus* larvae detected at inshore stations were always substantially less abundant than *M. edulis* larvae (percentage of total *Mytilus*; transect 1, cruise 2, 19%, cruise 7, 12%, cruise 8, 16%; transect 2, cruise 3, 5%; figure 1). By contrast, at offshore stations, *M. trossulus* larvae often, although not always, comprised a larger proportion of the *Mytilus* sample (transect 1, cruise 3, 11%, cruise 4, 22%, cruise 6, 40%, cruise 8, 13%; transect 2, cruise 3, 100%, cruise 5, 65%).

The distribution of *M. edulis* larvae was generally discontinuous across the shelf. On several occasions, larvae were either more abundant on the inshore ends of the two transects (transect 1, cruise 6, ANOVA, *F*=28.10, *p*<0.0001; cruise 7, ANOVA, *F*=9.46, *p*<0.005; post hoc tests for both reported in figure 6*b*; transect 2, cruise 4, ANOVA, *F*=15.52, *p*<0.0003; cruise 5, ANOVA, *F*=9.83, *p*<0.0006; post hoc tests for both reported in figure 7*b*), or at a cluster of stations on the inshore side of transect 2 (cruise 3, ANOVA, *F*=15.55, *p*<0.0001 and post hoc tests reported in figure 7*b*). On two cruises, high *M. edulis* abundance was observed at both transect ends or at clusters of stations at opposite transect ends (transect 2, cruise 6, ANOVA, *F*=7.73, *p*<0.005; cruise 7, ANOVA, *F*=10.85, *p*<0.0005; both post hoc tests reported in figure 7*b*). On other dates, maximum *M. edulis* abundance occurred near the offshore end of a transect (transect 2, cruise 8, ANOVA, *F*=40.26, *p*<0.0003 and post hoc tests reported in figure 7*b*), at an intermediate station (transect 2, cruise 2, ANOVA, *F*=39.00, *p*<0.0001 and post hoc tests reported in figure 7*b*), or in two maxima at the offshore end and at an intermediate station (transect 1, cruise 4, ANOVA, *F*=15.16, *p*<0.005 and post hoc tests reported in figure 6*b*).

## 4. Discussion

### 4.1 Nonlinearity of larval abundance estimates

The slight nonlinearity that we observed between known and estimated larval proportions in artificial mixtures (figure 5) was not surprising and has precedent based on work in other systems. Ribosomal DNAs are often used in PCR-based surveys of microbial community diversity. In such applications, biases in the per cent composition of a mixture of species that is estimated from a multi-template amplification may occur [56–58]. In microbial ecology applications, a degenerate, universal pair of primers (capable of amplifying from a phylogenetically broad range of microbial species) is often used in PCR and species-specific amplicons are distinguished by a downstream procedure, for example through restriction digestion in T-RFLP analysis [59,60]. Considerable bias has often been reported and stems from two broad classes of sources, termed PCR drift and PCR selection [61]. Drift occurs in early cycles of the PCR, is random and is not expected to be reproducible, whereas selection is a repeatable tendency for primers to amplify more efficiently off certain templates at the expense of others in the mixture. PCR selection is generally a consequence of primer site/template interactions [57,58] in which two templates differ in oligonucleotide sequence complementary to a primer and one template/primer hybrid molecule forms with higher affinity (e.g. due to higher GC content), and this template is then preferentially amplified. This explanation, however, cannot account for the reproducible bias in favour of the majority template in our mixtures, because primer site sequences in the 16S gene are identical across the two species, with identical mismatches to the universal primers we used (not shown). Moreover, the average GC content of the full-length *M. edulis* and *M. trossulus* 16S amplicons are similarly low (39.9% and 39.3%, respectively), so the thermodynamics of the two competing reactions are likely to be very similar.

The phenomenon responsible for the bias that we observed is probably unrelated to DNA sequence. But the bias against the rarer templates was reproducible—across multiple mixtures prepared, and with mixtures prepared from the offspring of two different haphazardly selected pairs of parents. It is possible that an unavoidable factor was responsible—low DNA concentration in our extracts—even though we went to considerable length to mitigate this problem by carefully cleaning up plankton samples and by using hot-start polymerase. Low DNA concentrations lead to a predictable drop in estimated diversity from multi-template PCR, due to the loss of rarer species in environmental samples [59,62] and loss of rarer alleles [63]: a form of molecular ‘sampling error’. In our application, however, this problem could not be overcome easily, as DNA yield was low and increasing it would have required processing very large water volumes (particularly when larvae were not abundant). It is possible that future studies could benefit from redesign of primers and a systematic study of bias, but for the present purposes our assay performed adequately. The slight bias against the rare species was easily estimated, and the minor adjustment for its presence had little effect on our results. Corrections to proportional abundance estimates were typically on the order of a few percentage points, while significant variation in abundance among stations involved differences of at least a factor of two and more often an order of magnitude (figures 6 and 7).

### 4.2 Across-shelf distribution of larvae

The across-shelf distribution of *M. edulis* larvae was consistent with our predictions based on the distribution of adults within the study region (figures 1 and 2) and expectations of very limited across-shelf mixing. On different sampling cruises and transects, *M. edulis* larvae were abundant on either the offshore or inshore ends of the transect, or in some cases, at both ends with abundance lower on mid stations (figures 6*b* and 7*b*). This range of distributions is consistent with larvae originating in either the EMCC or near-shore waters, or both, and limited mixing between the two sources.

*Mytilus trossulus* larvae were substantially less abundant than *M. edulis* larvae throughout the study region (figures 6 and 7, panel (*a*) versus (*b*)), which is perhaps indicative of conditions near a range boundary. As a consequence, the across-shelf patterns were less pronounced and more difficult to evaluate. Larvae of this species were often abundant on the offshore ends of the two transects (figures 6*a* and 7*a*), which is consistent with an origin in or entry to the region via the EMCC and limited mixing with the near-shore waters. In a few cases, *M. trossulus* larvae were also more abundant on the inshore end of a transect than at intermediate stations or the offshore end (transect 1, cruises 2, 7 and 8, figure 6*a*; transect 2, cruise 3, figure 7*a*). However, in all of these cases, congeneric*M. edulis* larvae were quite abundant and *M. trossulus* larvae constituted only 5–19% of the total *Mytilus* larval pool (cf. panels (*a*) and (*b*) in figures 6 and 7). This low relative abundance is comparable to adult abundance patterns inshore of the EMCC (figure 1) and so the larval distribution still appears consistent with a local origin (inshore of the EMCC). While the paucity of *M. trossulus* larvae means that the across-shelf distribution patterns provide only weak support for our central hypothesis of limited across-shelf mixing, those patterns also offer no strong evidence against that hypothesis. Furthermore, it appears that the *M. trossulus* range boundary may be more a function of a limited number of larvae entering the study region rather than the failure of those larvae to reach shore.

Although the eight cruises revealed evidence of limited across-shelf mixing of mussel larvae, physical mixing in this region is episodic [28] and the few observations reported here do not preclude sporadic across-shelf mixing events. Onshore transport of shelf waters is typically driven by downwelling [64] or offshore winds [3–5], depending on water depth and vertical stratification. Tilburg *et al.* [28] found that colder inshore temperatures, indicating onshore movement of the colder EMCC, occurred along transect 1 when winds were from 60 to 90°N and along transect 2 when winds were from 10 to 20°N. However, these wind events were not common, occurring approximately 6% and 2.2% of the time at transect 1 and 2, respectively [28]. Examination of winds at the National Oceanic and Atmospheric Administration’s Eastern Maine Shelf buoy (EB 44034) revealed that in 2011, wind events that would cause onshore movements of the EMCC were again rare, occurring less than 4% of the time. Although uncommon, these events could be expected to transport some larvae from the EMCC into the inshore region if larvae were present during these periods, as has been demonstrated on the California coast [2]. Larvae would then be expected to remain inshore of the EMCC due to the lack of across-shelf mixing.

The predominant southwesterly wind in this region should generally result in an upwelling regime in which surface water is advected offshore while EMCC water moves shoreward under the less dense near-shore water [5,65]. Fong *et al.* [66] examined the effect of upwelling winds in the WMCC and found that the offshore waters moved onshore and to the surface during upwelling wind events. Because we sampled mussel larval abundance at a single depth, we cannot exclude the possibility that mussel larvae originating in the EMCC are subducted under the near-shore water, where they might either migrate towards the surface or be transported into the surface water [64]. Although earlier field surveys reported mussel larvae only in the top few metres of the water column [45], subsequent to this study we have routinely retrieved them from as deep as 14 m in the Gulf ofMaine (Philip O. Yund 2012–2014, unpublished data), and bivalve larvae in general are common in the Northwest Atlantic down to 20 m or more [67]. Much smaller scale sampling around a river plume in the southwestern Gulf of Maine has provided evidence of this subduction mechanism [68]. However, we are sceptical that subduction results in across-shelf transport on the larger spatial scale of our study region. Because of the spatial scale involved, we were unable to constrain our sampling to a particular tidal phase or wind condition. But if *M. trossulus* larvae were both being subducted under the near-shore water and migrating or being transported upward to a 5 m depth, we should have detected more of them near shore. It is very unlikely that all of our sampling would have occurred while larvae from the EMCC were being subducted but had not yet migrated or been transported upward to our sampling depth.

### 4.3 Connectivity among *Mytilus edulis* populations

The EMCC divergence from shore has important implications for the connectivity of *M. edulis* populations. When *M. edulis* larvae were present, they were not homogeneously distributed across the shelf, but instead were more abundant on the offshore and/or inshore ends of our across-shelf transects (figures 6 and 7). Consequently, populations of *M. edulis* located inshore of the EMCC are not likely to be receiving an appreciable supply of larvae originating from populations to the northeast. By the same token, limited mixing across the EMCC/near-shore waters frontal boundary implies that larvae that originate inshore of the EMCC may be unlikely to disperse out into the EMCC and throughout the broader coastal current system. A logical consequence of this ecological barrier is some level of self-seeding in these near-shore populations, although our current work lacked sufficient spatial resolution to assess whether larvae are retained within individual bays or disperse among neighbouring bays. Either southwestward or northeastward larval dispersal is possible within the region inshore of the EMCC, depending on whether the small river plumes in the region create an inshore southwestward-flowing current that parallels the EMCC (such as the postulated Gulf of Maine coastal plume [35]), or larger scale processes such as high river discharge interacting with strong wind events [69,70] create a reverse flowing eddy shoreward of the EMCC.

Additional recent support for the isolation of bivalve populations inshore of the EMCC comes from a genetic study of sea scallops (*Placopecten magellanicus*), which reported that the single population sampled inshore of the EMCC (Gouldsboro Bay) was more highly differentiated from four other geographically widespread Gulf of Maine populations than any of those other four were from one another [71]. Although consistent with the scallop genetic results, our results probably do not extend to other invertebrate taxa that have larger and more strongly swimming larvae. For example, lobster (*Homarus americanus*) larvae are at least an order of magnitude larger than bivalve larvae (several mm versus 70–350 μm) and are very strong swimmers. The final lobster life stage prior to settlement (technically a post-larva, though it is nevertheless planktonic) is temperature sensitive and actively avoids colder water temperatures associated with the EMCC [72]. As a consequence, though lobster post-larval abundance is higher offshore than inshore in this region, settlement densities exhibit the reverse pattern, presumably as the result of active movement inshore [73].

The absence of mixing of larval bivalves between the coastal current system and near-shore waters reported for our study region is not likely to extend much further southwest of our study region. Depending on prevailing wind conditions, the EMCC either turns offshore at Penobscot Bay (approx. 70 km southwest of our study region), or returns to shore and merges into the WMCC [39]. The strong vertical stratification of the WMCC allows waters from the coastal current to move closer to the shore and mix with near-shore waters in the southwestern portion of the Gulf of Maine [74]. Consequently, bivalve larvae originating in or entering the coastal currents via the EMCC may be able to return to shore southwest of our study region. The combination of offshore topographic steering of the EMCC and inshore location of the WMCC may result in larvae that originate in the coastal currents settling both up- and downshelf of our study region, but not within the region itself.

Overall, our results highlight the need to better integrate studies of across-shelf mixing and larval dispersal into work on population connectivity [22]. Consideration of just the predominant along-shelf transport processes in our study region would lead to very different predictions for connectivity—i.e. rapid northeast to southwest transport with continuous exchange among populations along the coast [17,75]. However, a closer examination of the across-shelf transport mechanisms reveals that along-shelf variability in connectivity is not unexpected. Similar coastal circulation complexities exist along other coastlines [34], indicating that greater knowledge of those mechanisms that determine across-shelf larval transport is needed for a complete understanding of population connectivity.

## Data accessibility

Graphical representations of SST data are available from the University of Maine’s Satellite Oceanography Data Laboratory URL (http://wavy.umeoce.maine.edu/) and the raw data can be obtained by contacts provided on that site. Geo-referenced *M. edulis*/*M. trossulus* relative abundance data are provided in table form in Hayhurst and Rawson [37]. Per agreement with our funding agency (National Science Foundation, Oceanography Division), the metadata for the CTD and larval sampling presented here have been archived via the Biological-Chemical Oceanography Data Management Office (www.bco-dmo.org) as part of a broader project identified by the acronym MuLTI (‘Mussel Larva Transport Initiative’; http://www.bco-dmo.org/project/2138). The data associated with the subset of project metadata on which this paper is based will be released via this same portal promptly following publication.
